# Infectivity of DWV Associated to Flower Pollen: Experimental Evidence of a Horizontal Transmission Route

**DOI:** 10.1371/journal.pone.0113448

**Published:** 2014-11-24

**Authors:** Maurizio Mazzei, Maria Luisa Carrozza, Elena Luisi, Mario Forzan, Matteo Giusti, Simona Sagona, Francesco Tolari, Antonio Felicioli

**Affiliations:** 1 Department of Veterinary Science, Università of Pisa, Pisa, Italy; 2 Scuola Normale Superiore, Pisa, Italy; Salford University, United Kingdom

## Abstract

Deformed wing virus (DWV) is a honeybee pathogen whose presence is generally associated with infestation of the colony by the mite *Varroa destructor*, leading to the onset of infections responsible for the collapse of the bee colony. DWV contaminates bee products such as royal jelly, bee-bread and honey stored within the infected hive. Outside the hive, DWV has been found in pollen loads collected directly from infected as well as uninfected forager bees. It has been shown that the introduction of virus-contaminated pollen into a DWV-free hive results in the production of virus-contaminated food, whose role in the development of infected bees from virus-free eggs has been experimentally demonstrated. The aim of this study was twofold: (i) to ascertain the presence of DWV on pollen collected directly from flowers visited by honeybees and then quantify the viral load and (ii) determine whether the virus associated with pollen is infective. The results of our investigation provide evidence that DWV is present on pollen sampled directly from visited flowers and that, following injection in individuals belonging to the pollinator species *Apis mellifera*, it is able to establish an active infection, as indicated by the presence of replicating virus in the head of the injected bees. We also provide the first indication that the pollinator species *Osmia cornuta* is susceptible to DWV infection.

## Introduction

Honeybee viruses are pathogens which heavily contribute to the colony losses [1]. One of the most prevalent honeybee viruses is the Deformed Wing Virus (DWV) whose presence is generally related to infestation of the colony by the mite *Varroa destructor* [2]. DWV was first isolated in Japan in 1982 from deformed adult honeybees (*Apis mellifera L.*); to date its presence has been reported worldwide [3].

The virus belongs to the genus *Iflavirus* and its genome consists of a 10 Kb positive-strand RNA with a single open reading frame (ORF) flanked by a long 5′ untranslated region (5′ UTR) and a short, highly conserved 3′ UTR terminating with a 3′ poly-A tail [4]. DWV isolates from different parts of the world share 98 to 99% sequence identity [5], [6], consistent with the suggested recent global spread of the virus and a still limited evolutionary divergence [5]. The recent introduction of *Varroa* to Hawaii was followed by an increase in DWV infections and a decrease in DWV diversity suggesting selection for vectoring by *Varroa* as an additional factor contributing to the limited genetic variability so far observed among DWV isolates [7].

DWV can be transmitted vertically and persist in the bee colony as covert infection [8]. Overt infections, characterized by malformed or missing wings, shortened abdomens and premature death leading ultimately to the collapse of the bee colony [3], are usually associated with infestation of the apiary by *Varroa destructor* [9], [10]. The mite is not only able to support replication of DWV to high titers, which have been shown to correlate with the development of clinical signs in the bees [11], but also to activate latent infection in the bee via immunosuppression [8], [12], negatively regulating the expression of a member of the NF-kB gene family [13], which plays a central role in insect immunity [14].

DWV has been detected in many bee products, such as larval food, pollen and honey stored in the hive, suggesting that horizontal transmission can also occur through feeding and trophallaxis [15]. Consistent with this hypothesis, DWV-infected workers were shown to develop from DWV-negative eggs in the absence of *Varroa*, but in the presence of contaminated food [8], [16]. Additional evidence that the virus stored in bee-bread and honey was infectious was obtained by feeding virus-free colonies with these virus-contaminated foods, demonstrating that the queen became infected and laid infected eggs [17]. DWV was also found in pollen loads collected directly from uninfected forager bees, suggesting that the pollen-associated virus was previously laid on the flowers by infected pollinators [17].

The aim of this study was twofold: (i) to ascertain the presence of DWV on pollen collected directly from flowers visited by honeybees and then quantify the viral load and (ii) determine whether the virus associated with pollen is infective. To investigate the first issue we developed a highly sensitive TaqMan one-step qRT-PCR assay based on the RNA-dependent RNA polymerase gene of DWV and we analysed pollen samples collected from flowers visited by forager honeybees. To answer the second question we injected DWV suspensions obtained from visited flower pollen in two pollinator species, *Apis mellifera* and *Osmia cornuta*.

## Materials and Methods

### Honeybee and pollen samples

Symptomatic forager honeybees were collected from a *Varroa* mite infested apiary kept at the Department of Veterinary Science of Pisa University in San Piero a Grado (43°40′17″N–10°19′29″E), frozen and stored at −80°C until processed. In spring-summer 2012 pollen was sampled in the area surrounding the apiary. Pollen was directly gathered from sunflower (*Helianhus annus L.*), magnolia (*Magnolia grandiflora L.*) and ivy (*Hedera helix L.*) flowers both visited and unvisited by honeybees. The flowers which had to remain unvisited were covered with a net before blossom. Pollen loads were collected from foragers during their visit on magnolia and ivy flowers while the sunflower pollen load was obtained by brushing the foragers captured during their visit onto the sunflower flower-head (*capitulum*). Aliquots of 20 µg of each pollen sample were stored at −80°C until processed. Palynological analysis was performed on pollen load samples.

### Total RNA extraction from honeybee and pollen samples

Bee samples were homogenized using a TissueLyser II (Qiagen, Hilden, Germany) for 3 min. at 25 Mhz, total RNA was extracted with RNeasy Kit (Qiagen), eluted in 30 µl RNase-free water, quantified with RiboGreen RNA Quantitation Kit (Invitrogen, Carlsbad, CA, USA) and stored in aliquots at −80°C. Pollen samples (20 mg each) were suspended in 140 µl PBS, vortexed for 30 s and centrifuged at 14000 rpm for 5 min. Supernatant was used for RNA extraction with QIAamp Viral RNeasy Kit (Qiagen). RNA was eluted in 30 µl RNase-free water, aliquoted and stored at −80°C.

### DWV primers and dual-labelled probe

The complete genomes of DWV isolates of worldwide origin (GenBank ID: AY292384, NC_004830, GU109335, HM067437, HM067438, JQ413340, JX878304 and JX878305) and three sets of partial genomic sequences, belonging to DWV isolates of different geographical origin and coding respectively for structural proteins VP2-VP1, putative helicase and RNA-dependent RNA polymerase (Rd-Rp), were aligned with Clustal Omega [18] and the respective consensus sequences were used to design candidate sets of primers and TaqMan probes with Beacon Designer software v2.0 (Premier Biosoft International, Palo Alto, CA). The combination of primers and probe to be used in qRT-PCR assay was further selected based on the homology with the published DWV sequences.

Primers and probe amplified a 132 bp fragment within the highly conserved region coding for Rd-Rp. The sequences were: DWV Fw 5′- TTTGACATTGAGCTACAAGACTCG-3′ (nt. 8685–8708), DWV Rev 5′- ACAATCCGTGAATATAGTGTGAGG-3′ (nt. 8816–8793) and DWV probe Fw 5′- [6FAM]TCTCCTGCGTGGAATGCGTCCCGA [BHQ1]-3′ (nt. 8717–8740). Nucleotide positions here and throughout the paper refer to the DWV PA strain [4] [GenBank ID: AY292384].

### RNA standard

To generate the standard curve, 1 µg of total RNA extracted from a symptomatic bee was retrotranscribed with QuantiTect Reverse Transcription kit (Qiagen) using a blend of oligo-dT and random primers, according to the manufacturer's instructions. A 504 bp fragment encompassing the real-time amplicon was then amplified with HotStarTaq Polymerase (Qiagen) using 5 µl of cDNA as template with primer Fw 8450: 5′-TGGCATGCCTTG TTCACCGT-3′ (nt. 8450–8469) and primer Rev 8953: 5′-CGTGCAGCTCGATAGGATGCCA-3′ (nt. 8953–8932). The primers were designed with Primer-BLAST online software [19], based on the consensus sequence generated by alignment of DWV sequences available in GenBank. The amplified fragment was gel-purified, cloned into pCR 2.1 vector (TOPO TA Cloning kit, Invitrogen) and sequenced (BMR Genomics, Padova, Italy). The sequence was submitted to GenBank [GenBank ID: KF311109]. A plasmid clone was linearised downstream of the insert by BamHI enzymatic digestion and used as a template in a transcription reaction driven by the T7 promoter of pCR2.1 (MAXIscript SP6/T7 In Vitro Transcription Kit, Ambion, Austin, TX, USA). Following plasmid DNA removal by RNase-free DNase treatment (RNase Free DNase Set, Qiagen), the transcript was purified and concentrated with the RNeasy Minelute Cleanup Kit (Qiagen) and quantified with the RiboGreen RNA Quantitation Kit. The number of standard RNA molecules/µl was calculated and serial dilutions (2×10^5^ to 2 copies/µl) were prepared in RNA Safe Buffer [20].

### TaqMan qRT-PCR assay

A one-step assay was developed for the absolute quantification of DWV RNA. The assay was performed with Quantitect Probe RT PCR Kit (Qiagen) using a Rotorgene Corbett 6000 (Corbett Research, Australia). All reactions were carried out in duplicate, in a volume of 25 µl, with 10 µl of bee and pollen-derived RNA template and 5 µl of standard RNA. Primers and probe were used at a concentration of 0.4 µM and 0.2 µM respectively. The amplification programme was: 50°C for 30 min. to achieve reverse transcription, followed by 95°C for 15 min and then 50 cycles at 94°C for 20 s and 60°C for 1 min. Reactions without template were used as negative controls. To determine the linear range of amplification of the assay, a dilution series (2×10^5^ to 2 copies/µl) of standard RNA was used as a template and the reactions were carried out as described above. Bee sample results were expressed as viral copy number per microgram of RNA and pollen sample results as viral copy number per 20 mg of pollen.

### Strand-specific RT-PCR

A two-step RT-PCR was used for the specific detection of positive- and negative-strand DWV RNA. For each RNA two RT reactions were performed in a volume of 20 µl, in presence of the primer Fw 8450 or Rev 8953, with Superscript III reverse transcriptase (Invitrogen), following the manufacturer's instructions.

Briefly, incubation temperature was 55°C for 30 min, followed by 15 min at 70°C to stop the reaction and 20 min at 37°C in presence of RNaseH (Invitrogen, Carlsbad, CA, USA) to degrade the RNA. Five microliters of cDNA were used as template for the PCR reaction, which was carried out with HotStarTaqPlus Polymerase Mix (Qiagen), in presence of primers Fw 8450 and Rev 8953, which amplify a 504 bp fragment. A seminested PCR was carried out using 2 µl of the first PCR as template, with the qRT-PCR DWV Fw primer and the primer Rev 8953, to generate a 268 bp fragment. PCR products were analysed on a 2% agarose gel.

### Preparation of DWV suspensions

A total of 150 micrograms of pollen collected from sunflower, ivy and magnolia flowers visited by forager bees were pooled, resuspended in 500 microliters of phosphate buffered saline solution (PBS), vortexed for 30 s and centrifuged at 14000 rpm for 5 min. The same amount of pollen gathered from unvisited flowers of the same species was processed as described above and used as a negative control. Five grams of pollen load samples collected from forager bees at the entrance of the hive were suspended in 2.5 ml of PBS, vortexed and centrifuged as above. To prepare a positive control suspension, four infected honeybees were deprived of the poison sac, pooled, homogenized with TissueLyser II (Qiagen) for 3 min at 25 MHz in presence of 1 ml of PBS and centrifuged as above. The supernatants from visited and unvisited flower pollen (respectively VFP and UFP), pollen load (PL) and infected bees (IB) were sterilized by filtration through a 0.22 µm filter and stored at 4°C in the dark.

Viral RNA was extracted with Qiamp Viral Kit (Qiagen) from 210 µl of VFP, UFP and PL supernatants and from 140 µl of IB supernatant and eluted in 30 microliters of RNase-free water. Of each RNA 10 µl were used as template to determine the viral loads of the DWV suspensions by qRT-PCR.

The suspensions derived from visited flower pollen, pollen load and infected bees were shown to contain respectively 7×10^1^, 6×10^2^ and 1.9×10^8^ copies of DWV per microliter, resulting in 1.4×10^2^, 1.3×10^3^ and 3.8×10^8^ copies of virus being injected in each bee belonging to groups C, D and E respectively.

New viral supernatants, namely VFP1, PL1 and IB1, and UFP1 as a negative control, were prepared in summer 2014, with the procedure described above. The source of UFP1 and VFP1 were unvisited and visited sunflower flowers respectively. The PL1 and IB1 supernatants, which contained 3.6×10^3^ and 1.04×10^9^ DWV copies per microliter, were diluted to the same DWV level as VFP1, namely 6.8×10^1^ copies of DWV per microliter. Two microliters of VFP1 and diluted PL1 and IB1 viral suspensions, carrying 1.36×10^2^ copies of virus each, were used to inject the bees belonging to groups C1, D1 and E1 respectively.

### Injection assays on adult honeybees (*Apis mellifera*)

#### a. Assessing infectivity of flower pollen-associated DWV

Adult forager bees from a hive which was proven to be Varroa and DWV-free during the last two years [unpublished data] were selected for the injection experiment. Prior to the experiment twenty randomly chosen bees were analysed to confirm their status. To this purpose, the abdomens and the heads were dissected and homogenized with the TissueLyser II (Qiagen), the RNA was extracted as described above and analysed by qRT-PCR assay.

Fifty bees were divided into five groups, aA to aE. Group aA bees were not injected (negative control). Bees belonging to groups aB to aE were immobilized on ice and injected into the thorax haemolymph with 2 µl of UFP, VFP, PL and IB respectively. Following injection the bees of each group were kept for 10 days in small cages at room temperature and fed with a solution of 38% fructose, 32% glucose and 15% sucrose in water. Each day the dead bees were collected, frozen at −80°C, head and abdomen were dissected, soaked into RNAlater solution (Qiagen) and stored at −20°C until processed.

At day 10 p.i. the surviving bees were sacrificed and processed as above. The viral load in the head of control and injected bees was determined by qRT-PCR. Viral replication was assessed by strand-specific RT-PCR.

#### b. Comparing infectivity of DWV in VFP, PL and IB samples

A total of 210 nurse bees collected in august 2014 from the hive mentioned above were used in this experiment. Dissected heads and abdomens of thirty-five bees were analysed as previously described to ascertain their DWV status. The remaining 175 bees were divided into five groups, A1 to E1, each consisting of 35 bees. Group A1 bees were not injected (negative control). Bees belonging to groups B1 to E1 were injected with 2 µl of UFP1, VFP1, PL1 and IB1 respectively, and were kept and analyzed as described above, except that the last time point analysed was day 7 p.i. Statistical significance of the results was assessed using the Chi-Square test. P-values < 0.05 were considered statistically significant.

### Injection assays on adult mason bees (*Osmia cornuta*)

The DWV-free status of female mason bees of the species *Osmia cornuta* reared in an experimental breeding station of our Department was determined prior to the experiment: the abdomens and heads of twenty randomly chosen bees were dissected and the RNA extracted and analysed as described above. The injection experiment and the analysis of the viral load in the head and abdomen of control and injected bees (groups oA to oE) were carried out under the same conditions used for the honeybees.

## Results

### Palynological analysis

Pollen loads were collected from foragers during their visit on magnolia and ivy flowers while the sunflower pollen load was obtained by brushing the foragers captured during their visit onto the sunflower flower-head (*capitulum*).

**Figure 1:**
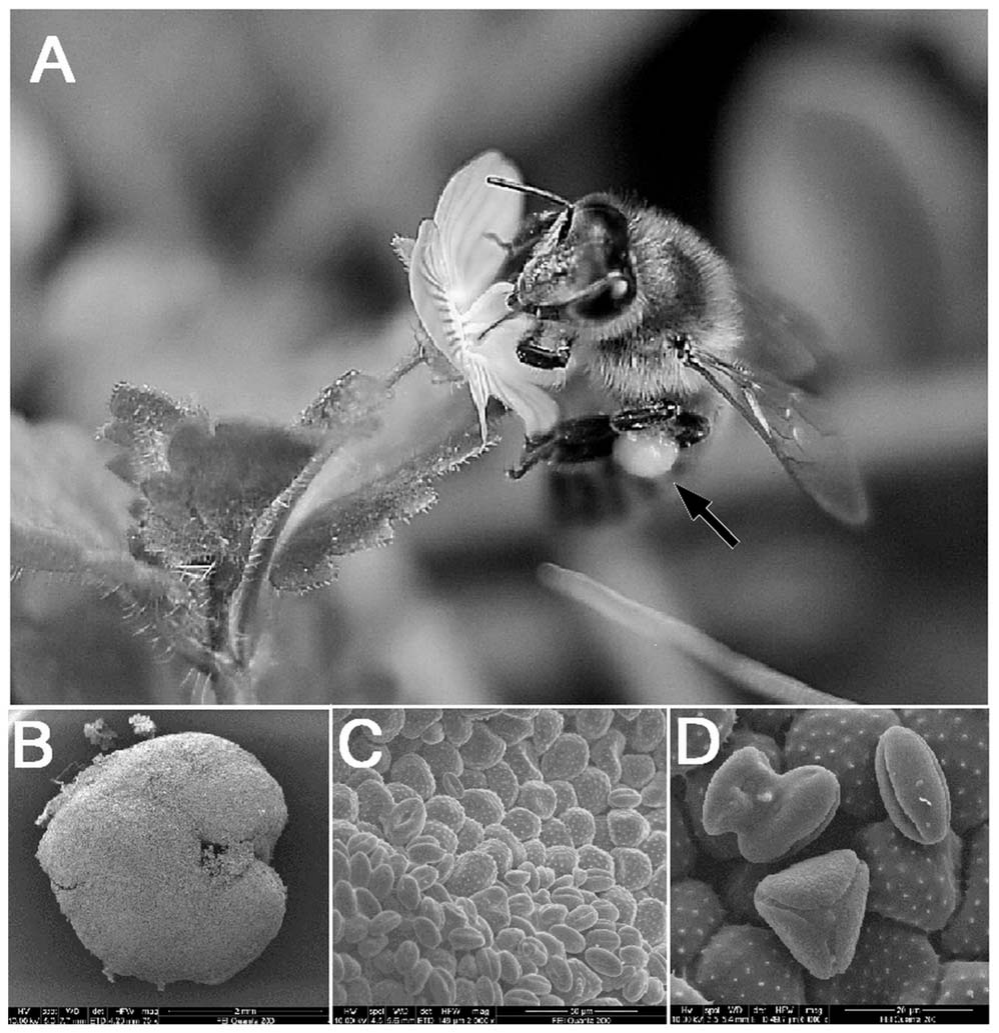
Foraging honeybee (*Apis mellifera* L.) and microscopic views of pollen. A) Pollen forager bee with pollen-load (black arrow); B) SEM micrograph of the entire pollen-load (70×); C) higher magnification of the pollen-load (2000×); D) pollen grains of a mixed-species pollen-load (6000×).

The palynological analysis showed that the pollen loads collected from forager bees captured while visiting magnolia, ivy and sunflower were mostly monoflora; some foragers were found to carry mixed species pollen loads (Figure 1).

### Linear range of amplification of DWV qRT-PCR assay

The linear range of amplification was determined using serial dilutions of the standard RNA, ranging from 1×10^6^ to 10 copies per reaction. The amplification of the standard dilutions showed linearity over five orders of magnitude, from 1×10^6^ to 100 copies of template RNA, with a lower detection limit of 10 RNA molecules per reaction.

### DWV viral load on pollen samples

**Figure 2:**
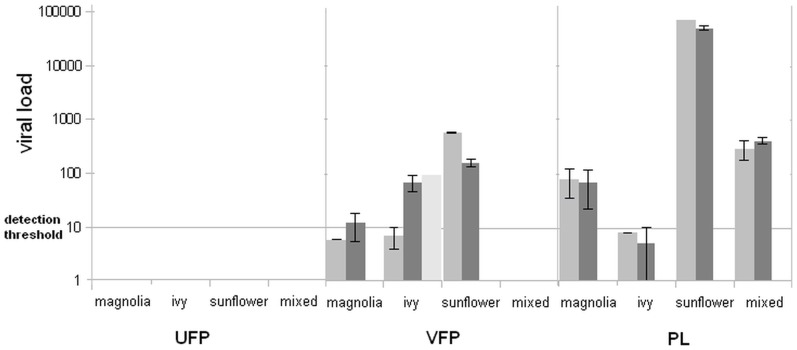
Viral loads of flower pollen and pollen load samples. The values are expressed as mean DWV copy number per 20 milligram of sample ± standar error (UFP: unvisited flower pollen;VFP: visited flower pollen; PL: pollen load).

Pollen samples collected from visited flowers harboured the virus, whereas the pollen from the unvisited flowers was virus-free. The DWV copy numbers determined on the magnolia, ivy and sunflower and on the respective pollen loads differed considerably, depending at least in part on the anatomy of the flower, the time of exposure to the pollinator's visits and the pollination behaviour of the forager bees. The results are shown in figure 2.

### Injection assays into adult honeybees

#### a. Assessing infectivity of flower pollen-associated DWV

In order to ascertain whether the virus present on visited flower pollen and on pollen load was infective, the DWV suspensions described above were injected into adult forager bees. To this purpose, twenty adult bees collected from a hive which was proven to be Varroa and DWV-free during the last few years by the qRT-PCR described above (unpublished results) were assayed to confirm their status prior to the injection experiment. Unexpectedly sixteen abdomens were weakly positive, with viral load values close to the detection limit of the assay (mean value 35.3±31.3 copies), whereas all heads were negative. In view of the observation that active infection is characterized by the presence of replicating virus in the head [15], [21], [22], it was decided to use these bees for the injection assays and to assess presence and replication of the injected virus exclusively in the head.

**Figure 3:**
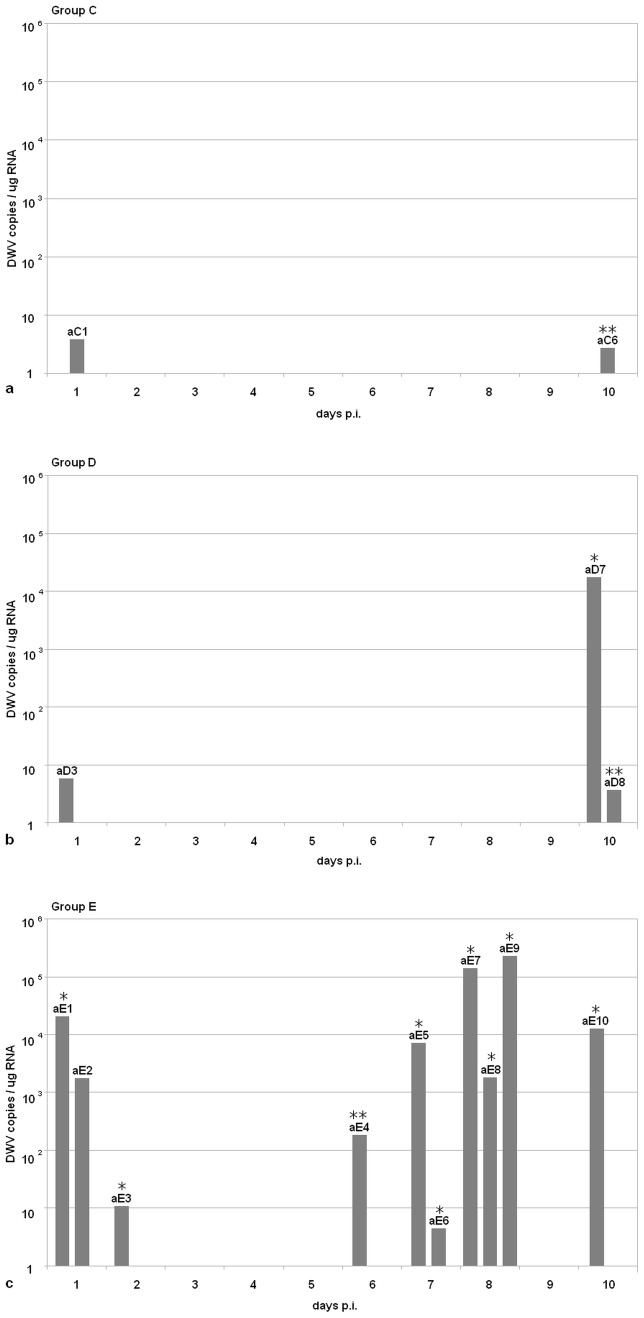
Infectivity of pollen-associated DWV. DWV-positive bees injected with VFP (a); PL (b); IB (c). X axis: days p.i.; Y axis: viral load in bee's heads; *: replicating virus detected after strand-specific RT-PCR; **: replicating virus detected after strand-specific seminested RT-PCR.

DWV RNA was found in the head of two bees injected with VFP supernatant, aC1 and aC6, which harboured few copies of viral genome (figure 3a). Three bees injected with PL supernatant, aD3, aD8 and aD7, were DWV positive; the first two harboured few copies of viral genome, whereas aD7 contained approximately 1.4×10^4^ DWV copies (figure 3b). All bees injected with IB supernatant, were positive, with most viral loads ranging from 3.6×10^2^ to 3.7×10^5^ DWV copies, with the exception of two bees, containing few copies of viral genome (figure 3c). The RNAs extracted from negative control bees, injected with UFP supernatant, as well as the RNAs of all uninjected bee's heads were negative.

#### b. Comparing infectivity of DWV in VFP, PL and IB samples

**Figure 4:**
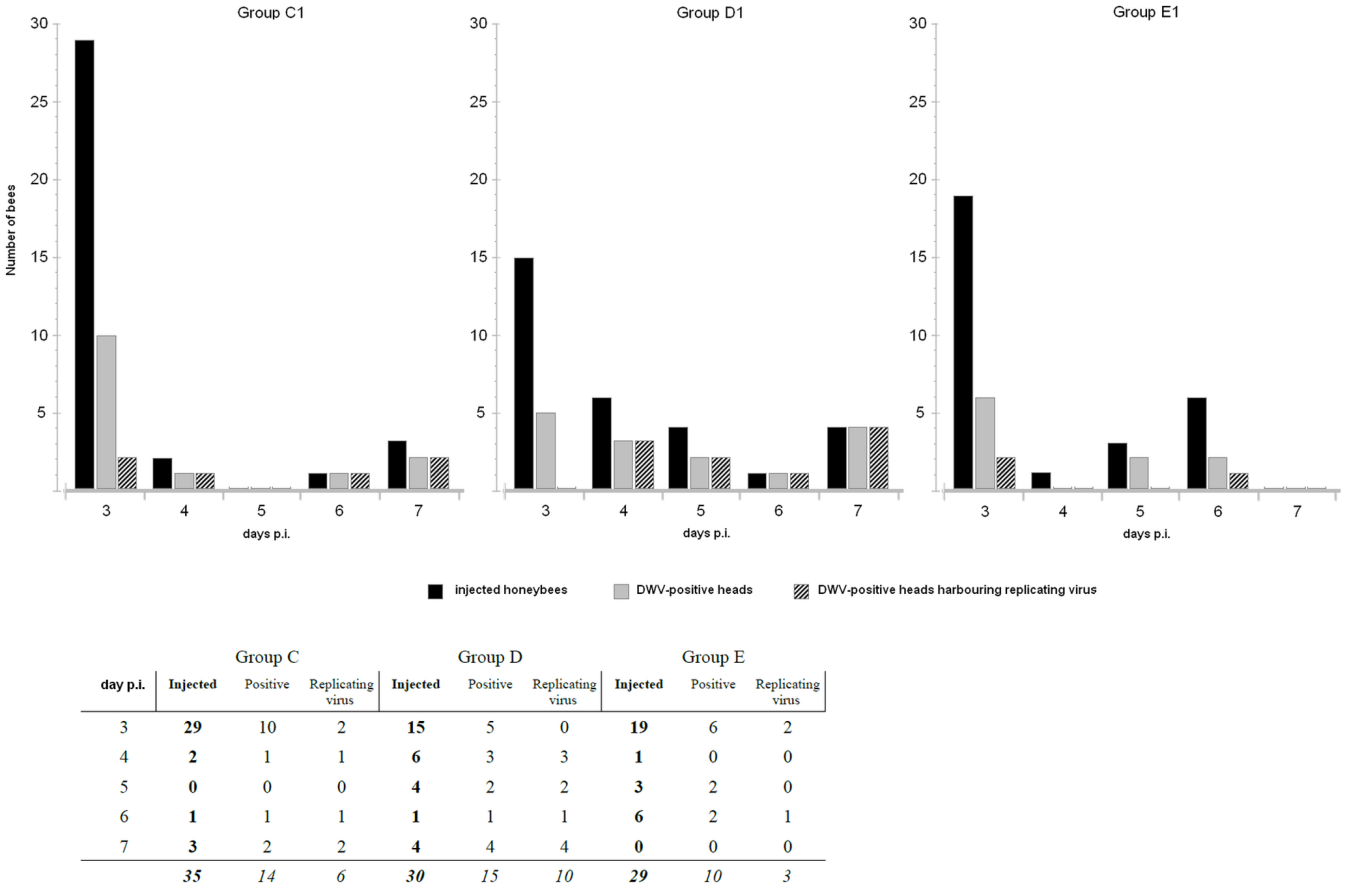
Comparison of DWV infectivity in VFP1, PL1 and IB1 supernatants.

Nurse bees were used in this experiment. Heads and abdomens of all bees analysed prior to the experiment to ascertain their DWV status were negative. A few bees within groups PL1 and IB1 died within few hours following injection and were not further analysed. No viral RNA was found in the heads of uninjected bees and bees injected with UFP1 suspension. DWV RNA was found in the head of fourteen out of thirty five VFP1-injected bees, in fifteen out of thirty injected with PL1 and in ten out of twenty-nine injected with IB1. Figure 4 shows the number of DWV positive heads of groups C1, D1 and E1 (gray bar) vs the total number of the injected individuals (black bar) at days 3, 4, 5, 6 and 7 p.i.

Significant difference was found between VFP1, PL1 and IB1-injected groups and the negative control group, with all p-values <0.01, whereas no significant difference was found between the infectivity of VFP1, PL1 and IB1 supernatants. The distribution of viral load values was broad irrespective of the group, with values comprised between 9×10^2^ and 1×10^4^ DWV copies at day 3, and between 1.9×10^2^ and 6.6×10^9^ at later time points, all well above the number of injected viral particles.

### Detection of replicating virus in injected honeybees

**Figure 5:**
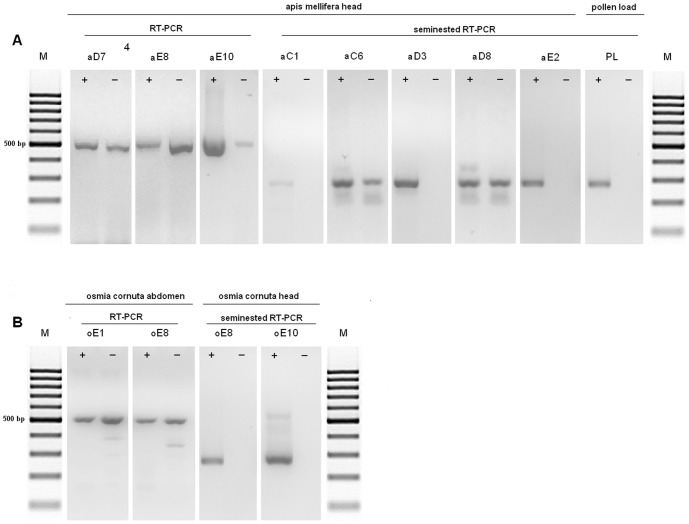
Detection of replicating virus by strand specific RT-PCR in injected *Apis mellifera* (A) and *Osmia cornuta* (B) bees.

DWV is a positive-strand virus, therefore negative-strand RNA is only found when the virus is replicating. A strand-specific RT-PCR assay demonstrated the presence of replicating virus in the head of one out of two positive honeybees injected with VFP, namely the one (aC6) which died at day 10 p.i. The head of bee aC1, which died 1 day p.i. only harboured positive strand RNA. Viral replication was found in two out of three positive bees injected with PL, namely aD7 and aD8, which were sacrificed at day 10 p.i., whereas bee aD3, which died at day 1 p.i. only carried positive strand RNA. All bees injected with IB had replicating virus, except one which died 1 day p.i. (Figure 5a). The RNA extracted from pollen load supernatant was used as a control for the specificity of the PCR; as expected, no replicative form of the virus was detected in this sample.

Similar results were obtained from the bees injected with VFP1, PL1 and IB1: at day 3 p.i. replicating virus was only detected in two positive heads of group C1 and two of group E1. From day 4 on, all positive heads of groups C1 and D1 harboured replicating virus, whereas E1 group positive heads only harboured negative strand RNA at day 6 (Figure 4, striped bars).

### Injection assays into adult *Osmia cornuta* bees

The abdomens and heads of twenty *Osmia cornuta* bees, analyzed prior to the injection experiment, were negative. The presence of DWV RNA was assessed in the head and abdomen of the injected bees belonging to groups B, C, D and E. DWV RNA was found in the abdomen of six bees, all belonging to group E, with most viral loads comprised between 5.1×10^2^ to 3.4×10^3^ copies. One of these bees (oE8) harboured viral RNA also in the head. One bee (oE10) harboured the viral genome exclusively in the head. The RNAs extracted from the bees injected with UFP supernatant, as well as the RNAs of all uninjected bee's heads were negative.

### Detection of replicating virus in injected Osmia bees

DWV was actively replicating in the abdomen of Osmia bees injected with IB, whereas no viral replication was detected in the two positive heads (Figure 5b). The RNA extracted from pollen load supernatant was used as a control for the specificity of the PCR; as expected, no replicative form of the virus was detected in this sample.

## Discussion

In this paper we provide quantitative evidence of Deformed Wing Virus association with pollen collected from flowers visited by forager bees (flower pollen) and we show that the virus associated with flower pollen is infective.

Our results strengthen the hypothesis that horizontal virus transmission among pollinators may occur via common visits to flowers [17], [23]. The TaqMan one-step qRT-PCR assay we developed targeted the highly conserved RNA-dependent RNA polymerase gene, with a linear range of amplification over 5 orders of magnitude and a sensitivity sufficient to detect few copies of DWV genome. The virus was readily detected on pollen samples collected from visited flowers of different species, with viral loads comprised between approximately 10 and few hundred copies. It must be emphasized that we did not measure the time of exposure of the flowers to the bee visits neither did we count the number of visits to each flower before the pollen was gathered. The viral particles were associated with the surface of the pollen grains as indicated by the observation that the virus was released into the supernatant after rinsing the pollen with PBS. This observation is consistent with previous results obtained on pollen pellets collected from forager bees [17]. Our data provide evidence that the virus is released on flower pollen by bees during their foraging activity. How the virus is transferred from the bee to the pollen is still unclear: it may likely occur via random deposition of virus-contaminated feces on the flowers [17], [24], as well as through contact between contaminated pollen load carried by the visiting bee and flower pollen. The virus-contaminated flower pollen samples identified in this study indicate that the virus is widely distributed in the environment outside the hive. Our finding along with previously reported experimental evidence of cross-species transmission of Israeli acute paralysis virus (IAPV) from honeybees to bumble bees via common visits to flowers [17], strongly suggests the role of pollen in the dissemination and transmission of DWV and other viruses. This transmission, which is believed to occur not only among honeybee colonies but also to other pollinators, is probably more relevant than previously thought.

It has been shown that the introduction of virus-contaminated pollen into a DWV-free hive results in the production of virus-contaminated food, whose role in the development of infected bees from virus-free eggs has been experimentally demonstrated [8], [17], [25]. Although the viral load resulting from the uptake and manipulation of virus-contaminated pollen may be low, it could however with time, via repeated ingestion by larvae as well as adult bees, lead to a covert infection of the colony. Infestation of such a colony by Varroa mites, regardless of the presence or absence of the virus within the mites themselves [26], will be likely to activate virus replication, via immunosuppression of the honeybee humoral and cellular antiviral immune responses [13], [27], leading to the onset of an overt, symptomatic infection.

However, it has not yet been elucidated whether the virus present on flowers, specifically on flower pollen, is infective, despite the exposure to potentially adverse environmental conditions.

To help elucidate this point we performed DWV injection experiments in adult individuals of two species, *Apis mellifera* and *Osmia cornuta*, which circulate and pollinate within the same area, sharing their foraging sites.

The *Apis mellifera* colony selected for the injection experiment tested DWV- negative by qRT-PCR during the last two years (unpublished results). When, shortly before the injection experiment, a control was performed on dissected heads and abdomens of a representative sample of the colony, all bee heads were negative, whereas most abdomens unexpectedly turned out to be weakly positive. Since it is widely accepted that active infection is characterized by the presence of replicating virus in the head [15], [21], [22], we decided to use this colony for the injection experiment and to assess presence and replication of the injected virus exclusively in the head. All *Osmia cornuta* individuals analysed before the experiment tested completely negative, allowing us to assess presence and replication of injected DWV both in the abdomen and in the head.

*Apis mellifera* and *Osmia cornuta* bees belonging respectively to groups aC and oC were injected with visited flower pollen supernatant while individuals belonging to groups aD and oD received supernatant from an enriched sample of pollen load collected from the foragers at the entrance of the hive. Two reasons determined this experimental choice. Firstly, Singh et al. [17] found DWV RNA in pollen loads of uninfected bees, as well as infected bees carrying uninfected pollen loads, and no obvious clustering of pollen load-derived viral sequences with the viral sequences found in the respective forager. These findings suggest that the virus found in pollen loads was most likely previously deposited on the flowers by infected pollinators. Secondly, the enriched pollen load inoculum carried tenfold more viral particles than the flower pollen inoculum, providing a higher possibility of success for the injection assay.

Injection of flower pollen supernatant produced detectable infection in the head of two *Apis mellifera* bees, with viral loads just above the lower limit of detection of our assay. This was not surprising, considering the very low number of viral particles injected (1.4×10^2^).

To this point it is important to mention that the number of viral particles used for this experiment was several orders of magnitude lower than previously shown to be necessary to reproducibly establish detectable DWV infection, i.e. DWV RNA signal in the head [21]. Although enriched tenfold with respect to the flower pollen inoculum, the pollen load inoculum carried only 1.3×10^3^ copies of DWV; nevertheless viral RNA was found in the head of three honeybees, of which one had died one day p.i. and two were sacrificed at day 10. Viral loads were low, except for one of the bees sacrificed at day 10, which carried 1.4×10^4^ copies of DWV genome, well above the number of copies present in the inoculum.

In agreement with the results of Mockel et al. [21], all bees belonging to group aE, which received 3.8×10^8^ virus particles, were DWV positive, with viral loads comprised between few units and 3.7×10^5^ copies of viral genome, irrespective of the time interval between injection and sampling. The strand-specific RT-PCR assay gave clear indication that the virus associated with flower pollen was actively replicating, as negative strand RNA was found in the head of bee aC6, sacrificed at day 10 p.i. No detectable replication was seen in the bee which died at day 1 p.i., a result which could be explained by assuming that this time interval was too short for the viral replication to produce a number of progeny genomes sufficiently high to be detected by our strand-specific nested RT-PCR. Similarly, viral replication was promptly detected in the head of bees aD7 and aD8, sacrificed at day 10 p.i., whereas only genomic RNA was present in bee aD3, which died at day 1 p.i. Replicating virus was found in most bees belonging to the positive control group aE.

The finding that visited flower pollen was infectious prompted us to perform an additional experiment with a twofold purpose: to assess the statistical significance of the results obtained in the first experiment, by injecting and analysing a higher number of bees and to compare the infectivity of DWV present in flower pollen, pollen load and infected bee-derived supernatants. To assess the statistical significance of the results obtained with these groups, UFP1-infected bees were used as a negative control group. Significant difference was found between all virus-injected groups and the negative control group whereas no significant difference was found between the infectivity of VFP1, PL1 and IB1 supernatants. In agreement with the results of the first experiment, after the first few days p.i., replicating virus, which is the hallmark of an active infection, was promptly detected in the head of the infected bees. These results confirm that DWV from flower pollen is able to establish an active infection in honeybee, irrespective of bee's age and of season, and strengthen the data about environmental stability of DWV [17] and of other picornaviruses exposed to sunlight, dessication and temperature fluctuations [28], [29].

Although Osmia bees were not infected by flower pollen under the experimental conditions used in this study, our results represent the first evidence that the species *Osmia cornuta* is susceptible to DWV infection, as shown by the evidence of viral replication occurring in the abdomen of the individuals injected with the IB extract.

In conclusion, our results indicate that deposition of DWV on flowers by infected forager honeybees may significantly contribute to the dissemination of the virus in the environment, representing a source of infection for other pollinators as well.
